# Evaluating the Risk of Suicide and Violence in Severe Mental Illness: A Feasibility Study of Two Risk Assessment Tools (OxMIS and OxMIV) in General Psychiatric Settings

**DOI:** 10.3389/fpsyt.2022.871213

**Published:** 2022-06-30

**Authors:** Gabrielle Beaudry, Manuel Canal-Rivero, Jianjun Ou, Jaskiran Matharu, Seena Fazel, Rongqin Yu

**Affiliations:** ^1^Department of Psychiatry, University of Oxford, Oxford, United Kingdom; ^2^Hospital Universitario Virgen del Rocío, Seville, Spain; ^3^CIBER de Salud Mental, Instituto de Salud Carlos III, Madrid, Spain; ^4^Instituto de Biomedicina de Sevilla (IBIS), Seville, Spain; ^5^Hunan Key Laboratory of Psychiatry and Mental Health, National Clinical Research Center for Mental Disorders, Institute of Mental Health, National Technology Institute on Mental Disorders, Central South University, Changsha, China

**Keywords:** suicide, violence, schizophrenia, bipolar disorder, risk assessment, prediction model, OxMIV, OxMIS

## Abstract

**Background:**

Two OxRisk risk assessment tools, the Oxford Mental Illness and Suicide (OxMIS) and the Oxford Mental Illness and Violence (OxMIV), were developed and validated using national linked registries in Sweden, to assess suicide and violence risk in individuals with severe mental illness (schizophrenia-spectrum disorders and bipolar disorders). In this study, we aim to examine the feasibility and acceptability of the tools in three different clinical services.

**Method:**

We employed a two-step mixed-methods approach, by combining quantitative analyses of risk scores of 147 individual patients, and thematic analyses of qualitative data. First, 38 clinicians were asked to use OxMIS and OxMIV when conducting their routine risk assessments in patients with severe mental illness. The risk scores for each patient (which provide a probability of the outcome over 12 months) were then compared to the unstructured clinical risk assessment made by the treating clinician. Second, we carried out semi-structured interviews with the clinicians on the acceptability and utility of the tools. Thematic analysis was conducted on the qualitative data to identify common themes, in terms of the utility, accuracy, and acceptability of the tools. The investigations were undertaken in three general adult psychiatric clinics located in the cities of Barcelona and Sevilla (Spain), and Changsha (China).

**Results:**

Median risk probabilities over 12 months for OxMIS were 1.0% in the Spanish patient sample and 1.9% in the Chinese sample. For OxMIV, they were 0.7% (Spanish) and 0.8% (Chinese). In the thematic analysis, clinicians described the tools as easy to use, and thought that the risk score improved risk management. Potential additions to predictors were suggested, including family history and the patient's support network. Concordance rates of risk estimates between the tools and clinicians was high for violence (94.4%; 68/72) and moderate for suicide (50.0%; 36/72).

**Conclusion:**

Both OxMIS and OxMIV are feasible and practical in different general adult psychiatric settings. Clinicians interviewed found that both tools provide a useful structured approach to estimate the risk of suicide and violence. Risk scores from OxMIS and OxMIV can also be used to assist clinical decision-making for future management.

## Introduction

Individuals diagnosed with severe mental illness (schizophrenia-spectrum disorders and bipolar disorders) are at an elevated risk for serious adverse outcomes, such as death by suicide and perpetration of violent crime (1–10). The lifetime risk of suicide is higher in people with schizophrenia than in the general public (around 5 vs. 1%) (1, 11). People with bipolar disorder also represent a high-risk group, with elevated suicide risk at least 10-fold to that of the general population (2, 3). Recent reviews have also reported evidence of an elevated risk of violence perpetration in severe mental illness (5, 8), with absolute risks of crime outcomes of up to 5% in women, and 25% in men with schizophrenia-spectrum disorders over three decades (8). Absolute risks of violent crime in bipolar disorders are also elevated (2% in women and 8% in men), with most crimes reported in the 5 years following diagnosis (5, 7).

Due to these increased outcome rates, risk assessment and management of suicide and violence are an integral part of clinical care in general and forensic psychiatric services. However, current approaches are inconsistent, and the relative importance of structured and unstructured psychiatric assessments of suicide and violence risk vary considerably across countries, services and clinical teams (12–16).

Most of the currently implemented tools are suboptimal in that they have at best moderate predictive accuracy (9). Further, prediction models are usually developed using limited data sources (e.g., only health), and often lack information on known risk factors due to data availability and inconsistent reporting (17). There is also a lack of consistency in risk definition and classification across instruments, and consequently, the rates of those deemed to be high risk vary considerably (18). Validation is rarely conducted outside of the study population in which tools are first derived (19, 20). Thereby, it is likely that some miscalibrated tools are currently employed to inform decisions along the clinical pathway (21).

One tool that aims to assess violence and suicidal outcomes is the Short-Term Assessment of Risk and Treatability (START)—a structured professional judgement (SPJ) tool designed to provide an estimation of short-term risk for a variety of adverse outcomes in mental health services, including self-harm, suicidality and aggression (22). The START includes 20 dynamic items in relation to both strengths and vulnerabilities, and also allows for the inclusion of additional clinical information. A meta-analysis of 543 patients from 2014 reported AUCs, but no other performance measures, for predicting violence (AUCs 0.52 to 0.89), but less evidence for self-harm (four AUCs ranging from 0.54 to 0.86) and no evidence on suicide (23). A randomized controlled trial suggested that the tool did not improve outcomes in clinical practice (24). In addition, the START has not been studied in any low- or middle-income countries with no external validations or feasibility studies (25), but there is one small validation in Japan (26).

Two OxRisk clinical prediction models—the Oxford Mental Illness and Suicide (OxMIS) and the Oxford Mental Illness and Violence (OxMIV)—were developed to address these methodological limitations (27, 28). Unlike structured clinical judgement instruments, these tools rely on routinely collected clinical information, and do not require additional assessment or subjective ratings from clinicians. This mitigates against variability amongst raters and subjective bias. Furthermore, they use risk factor information weighted by their association with later outcomes. National linked registries of around 75,000 individuals from Sweden were used to derive and validate the models, which achieved good calibration and discrimination. Calibration plots reflected adequate agreement of the predicted probability and observed proportions of suicide and violent offending outcomes. In external validation, the models showed good discrimination, as indicated by an overall *c*–index of 0.71 and 0.89 for OxMIS and OxMIV, respectively. Both tools incorporate routinely collected risk factors, some of which are modifiable for managing risk of suicide and violence. Other validation studies of OxMIV have examined different outcomes to the tool's primary outcome of 12 month violent crime in community settings. Even considering this, the tool performed moderately well. Specifically, in relation to inpatient violence in a forensic setting in Germany, an AUC of 0.72 was reported and for any interpersonal violence (i.e., not criminal) over 3 years in a research cohort in the Netherlands, it was 0.67 with calibration-in-the-large being adequate (29, 30). OxMIV is also more suitable to be used in general psychiatric hospital than other tools which require the presence of previous criminal offending in order to be administrated, such as VRAG/VRAG-R. As for OxMIS, a feasibility study in the UK found that natural language processing could be used to extract predictors from electronic medical records and that it was feasible to use in practice (31). The next step for research is to determine if OxMIS and OxMIV can improve clinical decision-making and assist in reducing adverse outcomes in clinical settings.

A recent systematic review (32) has found that very few studies have examined the impact of implementing existing prediction models to new clinical settings. Therefore, we conducted a feasibility study to examine whether OxMIS and OxMIV were acceptable and scalable to be used in general adult psychiatric settings in two different countries: China and Spain. The purpose of investigating samples from these two countries was to examine the feasibility of the tools in different geographical areas and cultural settings (East Asian vs. Western European country) and economies (upper-middle-income vs. high-income country). More specifically, the aim of this study was two-fold: to perform a quantitative assessment of the risk of violence and suicide in patients using the OxMIS and OxMIV tools; and to examine the acceptability of the tools and identify potential challenges for mental health professionals to use them routinely in their practice. Secondary objectives were to investigate the association between suicide and violence risk in patients, concordance rates between clinical judgement and risk scores, and potential differences in the use of these tools between countries.

## Methods

### Ethical Approval and Consent

This feasibility study was conducted in accordance with the Declaration of Helsinki. We received ethical approval from the institutional review boards of all three participating hospitals, namely the Second Xiangya Hospital of Central South University (Changsha, China), Hospital Universitario Germans Trías i Pujol (Barcelona, Spain) and Hospital Universitario Virgen del Rocío (Sevilla, Spain). Each study participant—both clinicians and patients—provided oral consent.

### Study Design, Procedures, and Participants

We employed a mixed-methods approach to evaluate the feasibility of using the OxMIS and OxMIV risk assessment tools in new clinical settings. Clinicians from three psychiatric hospitals, based in Changsha (China), Barcelona and Sevilla (Spain), were recruited using a convenience sampling method. We selected this sampling approach as data collection using other sampling methods, e.g., randomization, is often met with practical barriers and ethical issues in psychiatric hospitals (33–35). These clinicians were general psychiatrists (specialists), general practitioners (primary care physicians) and clinical psychologists with experience in conducting risk assessments of patients with severe mental illness. First, clinicians were asked to estimate the risk of suicide (within the next 12 months) for each patient on a continuum ranging from 0.5% (marked as being the average) to 5%. Second, each clinician was also asked to calculate the probability risk scores for both tools (using an online risk calculator at http://oxrisk.com), in addition to their routinely conducted risk assessments. Consenting patients were included if they were aged 18 years and older, and had a formal diagnosis of schizophrenia-spectrum disorders or bipolar disorders (as defined by the Diagnostic and Statistical Manual of Mental Disorders [DSM-5] or the International Classification of Diseases 10th Revision [ICD-10]).

Following this, clinicians were asked to complete semi-structured interviews by means of two questionnaires (one for each tool) to evaluate the utility and acceptability of the tools. These standardized questionnaires were developed and previously used to evaluate the feasibility of implementing OxMIS and another risk assessment tool FoVOx (Forensic Psychiatry and Violence Oxford) in clinical settings (31, 36). The OxMIV semi-structured interview questionnaire for clinicians was designed prior to the OxMIS one, and thus some items differ between the two questionnaires. Questions were based on the impact, accuracy, practicability and simplicity of the tools via multiple-choice (e.g., rating various characteristics of the tools), short answers (e.g., identifying facilitators and barriers to using the tools), and Likert scale formats (e.g., not at all, slightly, somewhat, very or extremely similar). Their answers were subsequently translated into English by members of the research team who were fluent in English and either Mandarin or Spanish. Any confidential information about patients was anonymised.

Data collection took place from 2019 to 2021 across all three research sites. Specifically, quantitative and qualitative data were collected between May and December 2019 (Changsha), January and February 2020 (Barcelona), and June and July 2021 (Sevilla).

### Sample Size

There are currently no explicit numerical recommendations for sample size in feasibility studies, or even qualitative health research (37), in contrast to external validation studies (38–40). We followed a sample saturation principle to identify and select participants related to study aims. This approach is that past a certain point, further data collection is unnecessary, as it only leads to data redundancy (41). Guidelines for feasibility studies recommend using a minimum sample size of ten (42), and thus our sample size of 38 clinicians was sufficient.

### Risk Factors

Predictors in OxMIS and OxMIV can be broadly grouped into three categories: sociodemographic, clinical and familial factors (see Supplementary Material). We employed the same definitions as those used in the original derivation studies (27, 28). Data on risk factors were obtained from existing medical records, and supplemented by verbal information collected during clinical consultations.

### Outcomes

OxMIS and OxMIV were first developed to estimate the risk of suicide and new violent offending within 1 year, respectively. In the derivation studies, suicide included death by intentional self-harm and undetermined deaths (i.e., ICD codes X60–84 and Y10–Y34) consistent with previous research (27, 43), and violent offending was defined as any act of physical and sexual violence against others, including illegal threats and harassment, that resulted in a conviction (28). Defining violent crime as conviction, rather than arrest, reduces the possibility of systemic biases in the ascertainment of violence amongst people with severe mental illness. However, violence being defined as conviction in the derivation study does not mean that it cannot be used to predict other forms of violent behavior, such as interpersonal violence based on clinical records and self-report. In fact, the tool has been validated for these non-crime outcomes (29). Thus, our primary outcomes were patient information on the relevant risk factors, OxMIS and OxMIV risk scores for each patient, suicide risk estimation of clinicians (in relation to OxMIS), and their responses to the semi-structured interviews.

### Analytic Strategies

The qualitative data was analyzed by two researchers (GB and JM) scoring independently. Using a thematic analysis approach, themes and subthemes were generated, and then compared between researchers to identify common findings. Any discrepancies were resolved by discussion with a senior researcher (RY). We used NVivo V.12 Software for qualitative data analysis (44).

In the quantitative analyses, the frequency or the median and interquartile range were calculated for each risk factor, as appropriate. Missing information was recorded as ‘unknown,’ which allowed for the calculation of the estimated risk score range. We reported the proportion of missing data for each predictor. Risk scores were calculated for each patient to estimate the probability of experiencing the two outcomes. Primary analysis was conducted to evaluate the proportion of patients in each risk category for suicide and violence, and compare them between tools and countries using Spearman's rank order correlation coefficient (Spearman's rho). Suicide risk, as measured by OxMIS, was grouped into three numerical categories (i.e., low [ <0.5%], moderate [where the range of risk scores for an individual included 0.5%], and increased [>0.5%]). This is a lower threshold than in the original OxMIS study and based on a pilot work estimating mean risk scores (31). For OxMIV, the original risk categorization was employed, and subsequently translated into violence risk levels (i.e., low [ ≤5%] or increased [>5%]). This threshold was selected based on the incidence of violence (following initial diagnosis) based on longitudinal research (6). We calculated the concordance rates for suicide by comparing clinical judgement with the risk categories based on OxMIS. To supplement this, we also calculated Spearman's rho between the clinician's own predicted risk value with OxMIS in the Chinese sample (which was not possible in the Spanish sample due to no individual level identifiers) (45). Such analysis could not be undertaken for violence risk due to no linkage information at patient level.

### Role of Funding Sources

The funders of the study had no role in study design, data collection, data analysis, data interpretation, or writing of the report. All authors had full access to the data in the study and had final responsibility for the decision to submit for publication.

## Results

### Sample Characteristics and Risk Factors

The characteristics of the Chinese and Spanish samples are reported in Table 1. In total, data from 147 patients were analyzed. More specifically, risk factors were extracted for 60 patients from China and 87 patients from Spain. There were some differences in the Chinese and Spanish samples in sex distribution and mean age. For patients with missing information on predictors, minimum and maximum values of coefficients were adopted to provide an estimated risk probability score (range of values). The mean of this range of values was used in correlational analyses for individuals with missing information.

**Table 1:** Distribution of risk factors for suicide (OxMIS) and violence (OxMIV) in severe mental illness in patient samples from China and Spain.

**Risk factors**	**China (*n =* 60)**	**Spain (*n* = 87)**
***Common variables***		
**Age**	23 (19–32)	41 (27–53)
**Sex**		
Male	21 (35.0)	41 (47.1)
Female	39 (65.0)	46 (52.9)
**Previous violent crime**	5 (8.3)	7 (8.1)
**Previous drug misuse**	1 (1.7)	36 (41.4)
**Previous alcohol misuse**	1 (1.7)	32 (36.8)
**Previous self-harm**	14 (23.3)	39 (44.8)
**Highest education**		
Secondary	17 (28.3)	38 (43.7)
Upper secondary	18 (30.0)	16 (18.4)
Post-secondary	23 (38.3)	27 (31.0)
Unknown	2 (3.3)	6 (6.9)
**Parental drug or alcohol misuse**		
Yes	0 (0.0)	20 (23.0)
No	49 (81.7)	60 (69.0)
Unknown	11 (18.3)	7 (8.0)
**Recent antipsychotic treatment**	56 (93.3)	49 (56.3)
**Recent antidepressant treatment**	26 (43.3)	58 (66.7)
**Current episode**		
Outpatient	0 (0.0)	63 (72.4)
Inpatient	60 (100.0)	24 (27.6)
**Benefit recipient**	0 (0.0)	32 (36.8)
***OxMIS–specific variables***		
**Length of first inpatient stay**		
≤7 days	10 (16.7)	59 (67.8)
>7 days	50 (83.3)	28 (32.2)
**Number of previous episodes**		
≤7 episodes	57 (95.0)	83 (95.4)
>7 episodes	3 (5.0)	4 (4.6)
**Parental psychiatric hospitalization**		
Yes	0 (0.0)	6 (6.9)
No	60 (100.0)	79 (90.8)
Unknown	0 (0.0)	2 (2.3)
**Parental suicide**	0 (0.0)	2 (2.3)
**Comorbid depression and schizophrenia**	3 (5.0)	15 (17.2)
***OxMIV–specific variables***		
**Parental violent crime**		
Yes	0 (0.0)	5 (5.8)
No	51 (85.0)	77 (88.5)
Unknown	9 (15.0)	5 (5.8)
**Sibling violent crime**		
Yes	1 (1.7)	8 (9.2)
No	52 (86.7)	74 (85.1)
Unknown	7 (11.7)	5 (5.8)
**Recent dependence treatment**	0 (0.0)	22 (25.3)
**Personal income**		
1st decile	14 (23.3)	1 (1.1)
2nd decile	3 (5.0)	5 (5.74)
3rd decile	3 (5.0)	36 (41.4)
4th decile	1 (1.7)	0 (0.0)
5th decile	2 (3.3)	37 (42.5)
6th decile	4 (6.7)	2 (2.3)
7th decile	0 (0.0)	6 (6.9)
8th decile	0 (0.0)	0 (0.0)
9th decile	0 (0.0)	0 (0.0)
10th decile	0 (0.0)	0 (0.0)
Unknown	33 (55.0)	0 (0.0)

*Data are median (IQR) or n (%). The updated version of OxMIV has the personal income variable changed to two categories (i.e., low and stable)*.

We recruited a total of 38 clinicians (26 from China and 12 from Spain), the majority of which were general psychiatrists (86.8%). Most Chinese clinicians worked in intensive care units (61.6%) and psychiatric inpatient settings (general adult wards [53.3%]). Spanish clinicians were also based in inpatient settings (58.3%) and others were in outpatient settings (41.6%). The number of assessed patients per clinician also varied between countries, with most clinicians in China evaluating 1–4 patients (96.2%), and those in Spain assessing 5–9 (41.6%) and 10–20 patients (58.3%). Some Chinese clinicians reported using another tool or process for suicide (36 [60.0%] out of 60 cases) and violence risk assessment (23 [38.3%] out of 60 cases). Similar figures were found in Spain for suicide risk assessment (5 [41.6%] out of 12). However, none of the Spanish clinicians previously used a tool to evaluate violence risk (see Supplementary Table 1 for list of tools used).

### OxMIS and OxMIV Scores

OxMIS and OxMIV probability risk scores were calculated with information from medical records and clinical consultations. As estimated by OxMIS, the median probability of suicide within 1 year was 1.9% (range = 0.6 to 10.3%) in the Chinese sample and 1.0% (range = 0.2 to 12.5%) in the Spanish sample. Based on a pre-specified threshold of 0.5% to determine risk categories (i.e., low, moderate, and increased), most Chinese patients were at increased risk (*n* = 59, 98.3%). None were low risk, and one moderate. In the Spanish sample, 21 (24.1%) were categorized as low risk, two at moderate risk (2.3%), and 64 at increased risk (73.6%).

Using OxMIV, the median probability of violence within 1 year was 0.7% (range = 0.2 to 14.4%) in the Chinese sample and 0.8% (range = 0.0 to 30.2%) in the Spanish sample. Most patients in China (55 [91.6%]) were categorized as low risk according to a pre-specified cut-off (<5.0%). One individual was at moderate risk (range of estimated values included 5.0%) and 4 others were at increased risk (>5.0%). As for the Spanish sample, 83 patients (95.4%) were categorized as low risk and 4 (4.6%) at increased risk for violence.

We found a medium effect size for the correlation of the risk of suicide and violence in the Chinese sample, *r*_*s*_ (46) = 0.45, *p* < 0.001. Amongst the patients from Spain, a small effect size was found for the correlation between the risk of suicide and violence, *r*_*s*_ (85) = 0.23, *p* = 0.03.

### Concordance Between OxRisk Tools and Clinical Judgement

In the semi-structured interviews, clinicians from Spain completed one questionnaire each (*n* = 12), whereas those from China completed one questionnaire per patient (*n* = 60). Half of clinicians described the OxMIS score as an accurate representation of risk (50.0%; 36/72), and almost all found that OxMIV compared similarly to their clinical judgement of risk (94.4%; 68/72). Overall, 41.7% of clinicians (30/72) reported that OxMIV had an impact on their clinical practice. A summary of the clinicians' detailed viewpoints in regards of OxMIS and OxMIV is provided in Tables 2, 3. Results from the multiple-choice items are presented in the Supplementary Material (Tables 2, 3). In addition, there was a significant correlation between the OxMIS risk score and clinical judgement, *r*_*s*_ (58) = 0.51, *p* < 0.001.

**Table 2:** Detailed viewpoints of clinicians on practicality and future use of OxMIS.

**Country**	**Theme**	**Sub-theme**	**Examples**
China	Clinical benefits	Assessment	Additional dimensions and information to assess patient risk; can be used to assist in evaluating the status quo of patients; provides a more comprehensive understanding of the risk of violence in patients; improves the efficiency of clinical work; suitable for a simple initial review; allows for consideration of different perspectives when evaluating patient risk; pay more attention to patients' past life events and familial factors
		Management	Provides a reliable reference value which is helpful for risk management with patients; can improve interventions for patients' impulsive behavior and strengthen psychological counseling when patients are at high risk score; knowing the risks, family members outside the hospital can also participate in active management; more detailed dynamic observation
		Interpretation	Interpretable and referential; can be used as a clinical reference; more in-depth understanding of the patient's psychological and emotional state
		Prevention	Improves prevention of suicide risk for patients; if the score is high, clinician can focus on the prevention of suicidal self-injury and other related behaviors; being more alert to suicide risk in patients
	Potential improvements	General limitations	No clinical judgement was considered; short-term risk is not considered in clinical judgement and long-term scale is difficult to evaluate; limited assessment information; comprehensiveness of the content needs improvement; sensitivity and applicability of the tool in Chinese population requires verification
		Relevance of existing risk factors	Being a benefit recipient (i.e. “income from welfare”) is not applicable to the Chinese context
		Risk factors not currently identified	Include situational questions (e.g. recent negative life events); lack of clinical symptom entries and insufficient attention to etiology; recent negative life events may also affect a patient's risk of suicide; influence of environmental factors
		Static nature of OxMIS	Patient's condition is variable and susceptible to life events; fluctuations are difficult to control; patient's life events and illness fluctuations also account for a certain proportion of the suicide risk; not necessarily consistent with the patient's current state
Spain	Clinical benefits	Assessment	Assessing risk more accurately; more extensive assessment of affective symptoms and suicidal ideation; allows for a more cautious evaluation of risk
		Management	Useful to make clinical decisions
		Interpretation	Considering risk in a quantitative manner allows for an approach that is tailored to each patient's personal risk
		Prevention	Without quantitative markers or prediction tools in clinical practice [like OxMIS], it is difficult to assess the most efficient strategies for prevention
	Potential improvements	Relevance of existing risk factors	Include specific timeframe for some predictors (e.g. parental psychiatric hospitalization 30 years ago might not be a good predictor)
		Risk factors not currently identified	More questions about clinical state (such as suicidal-related behavior over the past year); other situational and contextual factors (e.g. loneliness and degree of family support)

**Table 3:** Detailed viewpoints of clinicians on practicality and future use of OxMIV.

**Country**	**Theme**	**Sub-theme**	**Examples**
**China**	Clinical benefits	Assessment	Better grasp and pay attention to clinical practice; understand and consider the influence of family history and past history on patients
		Management	Moderate treatment, reduce usual vigilance for impulsive behavior
		Prevention	Objectively reflecting on the risk of violent incidents; interview process is helpful for clinicians to ensure their own safety; also considers whether patients are at high risk of harm to self; increase the prevention level for violent impulses
	Potential improvements	General limitations	Needs to be more in line with clinical reality; tool is not officially in the medical system
		Risk factors not currently identified	More related clinical entries; pay attention to the patient's current symptoms; consider the mental health of the patient's family members; welfare income; recent stressful events
**Spain**	Clinical benefits	Management	Useful to discuss with the entire team about some specific risk factors
		Prevention	Be more aware of the violent behavior risk
	Potential improvements	General limitations	Some information is difficult to obtain
		Risk factors not currently identified	Agitated behavior; more information about personal relationships with others

### Overall Views on Practicality and Future Use

We identified common themes from detailed qualitative analysis of clinician semi-structured interviews about OxMIS and OxMIV (see Tables 2, 3 for summarized qualitative results). Their overall views on the practicality and future use of the tools were grouped into two broad themes: clinical benefits and potential improvements.

Clinicians underscored improved risk assessment, management, interpretation, and prevention as clinical benefits of incorporating these tools in their practice. They also described potential additional predictors and some overall issues. For OxMIS, these included general limitations, relevance of existing risk factors, others not currently included, a lack of repeated measures over time, and accuracy. Sub-themes of potential improvements for OxMIV also included general limitations and risk factors not currently identified.

In terms of practicality, all Spanish clinicians and most Chinese ones (for 49 [81.6%] out of 60 cases) stated that the OxMIS web-based calculator would be practical to use as part of a suicide risk assessment or treatment plan. A majority of clinicians indicated that they would consider using OxMIS in the future (88.8% overall; 90.0% in China and 83.3% in Spain). For OxMIV, very few clinicians reported having encountered practical barriers in their local clinical setting that limited its use (6.9%; 6.6% in China and 8.3% in Spain). Only one clinician from Spain said that it was “not at all likely” for them to use OxMIV in the future. The remaining responses ranged from slightly to extremely likely.

## Discussion

We report the results of a feasibility study of two scalable evidence-based risk assessment tools, OxMIS and OxMIV, which have online risk calculators to enable their translation into routine practice. In general psychiatric services from two different countries, China and Spain, we estimated suicide and violence risk. Overall, we examined 147 patients and interviewed 38 clinicians. Risk probabilities generated by the clinical prediction models were compared quantitatively and qualitatively with clinical judgement. Semi-structured interviews were conducted with clinicians to assess acceptability and potential utility of implementing these tools in clinical practice.

The qualitative analyses indicated that clinicians found it was feasible and acceptable to use OxMIS and OxMIV for suicide and violence risk assessment at different points in the clinical pathway. They reported clinical benefits including improved risk assessment, interpretation, management and prevention of these two adverse outcomes amongst patients with severe mental illness. Qualitative feedback underscored the lack of consensus about what existing tools to use or processes for suicide and violence risk assessment in the participating hospitals, suggesting the need for simple and scalable prediction rules—a gap that could potentially be filled by OxMIS and OxMIV. Clinicians suggested improvements to these tools, mostly focusing on the inclusion of additional situational and contextual risk factors (e.g., loneliness, degree of family support, or recent negative life events).

Concordance between OxRisk tools and clinical judgement was high. Most clinicians described the OxMIS and OxMIV as accurate representations of risk of suicide (50.0%) and violence (94.4%), respectively. The former was consistent with the high correlation between the OxMIS risk score and clinical judgement, *r*_*s*_ (58) = 0.51, *p* < 0.001. We could not calculate this correlation for the risk of violence, as this information was not included in the OxMIV questionnaire. However, future feasibility studies could add this clinical estimate of risk.

Other important findings are inter-country similarities and differences. We found similar estimates of violence risk as measured by OxMIV, with 91.6% of Chinese patients and 95.4% of Spanish patients categorized as low risk based on pre-specified thresholds. With regard to the OxMIS tool, most patients in China (98.3%), and ~3 out of 4 (73.6%) patients in Spain were at increased risk for suicide. Due to difficulties in accessing mental health services and high levels of stigma toward mental illness in China (47), it is likely that the Chinese sample included more severe cases of schizophrenia and bipolar disorder than the Spanish sample. It is therefore possible that this contributed to the suicide risk difference between the two samples.

China and Spain differed in the correlation between the risk of suicide and violence, as estimated by the tools. While a positive correlation was found in both samples, the association was stronger in the Chinese sample than in the Spanish sample [*r*_*s*_ (58) = 0.45, *p* < 0.001 vs. *r*_*s*_ (85) = 0.23, *p* = 0.03]. A possible explanation for this result may be that patients in the Chinese sample were more severely ill. All patients from China were recruited from inpatient settings, whereas around a quarter of Spanish patients were inpatients at the time of the study. It is therefore possible that the link between violence and suicide is stronger in inpatients than outpatients. There is evidence of heightened suicide risk following admission and discharge (48) and some research suggests that being subjected to threats and assaults is more frequent in inpatient than outpatient settings (49). Moreover, the inclusion of eight patients from a secure unit within one of the two general psychiatric clinics in China could also have played a role in this association. Another potential explanation could be a larger treatment gap for mental health problems in China (50, 51), leading to those in China with a mental disorder who manage to access professional help having a more severe presentation than people with similar disorders in Spain.

### Limitations and Future Research

Limitations include selection of patients and clinicians, which reflected a convenience sample. While this is a valid approach, particularly when conducting research with individuals that can otherwise be hard-to-reach, some studies caution against generalizing findings due to the potential risk of selection bias (35, 52). We did not conduct an external validation of OxMIS and OxMIV as this was not the aim. Future research could consider this, and updating of the predictor coefficients may be required (53). Risk categories may also need to be adjusted. However, our findings are consistent with that of previous studies of OxMIS and OxMIV in which most participants were categorized at increased risk of suicide, but low risk of violence (29–31). Using an inter-rater design to assess the reliability of the tools should also be considered for future studies.

Further work is also necessary to establish whether OxMIS and OxMIV improve clinically relevant outcomes, such as rates of future adverse events and cost effectiveness of implementing these tools. Impact analysis is one recommended method to evaluate the real effect of a prediction rule on a given clinical setting. This could take the form of a multicenter randomized controlled trial (RCT)—with hospitals or clinics being assigned randomly either to the use of the prediction rule or conventional risk assessment practice. Patients would then be followed up to determine the impact of the tool on the outcome of interest (e.g., suicide or violence) (54). The feasibility of such studies will need to be determined.

Qualitative feedback regarding country-specific aspects highlighted one or two predictors that might not translate well to China. One such example is benefit recipiency in China, of which the prevalence was nill for OxMIS. This result was corroborated in the interviews with clinicians who suggested that this predictor was not applicable. Despite recent progress in welfare system reforms, social safety nets in China remain relatively weak when compared to other economically developed countries (55). As benefit recipiency is one of the weakest predictors in OxMIS, this is unlikely to have any material impact on the tool's accuracy, and could be removed altogether for Chinese populations. Other research could be undertaken to establish if there is a proxy measure that is associated with risk.

Further, the prevalence of both previous drug and alcohol misuse, and parental drug or alcohol misuse was particularly low in the sample of patients from China compared with Spain. Taken together, stigmatization against people with substance use disorder (56), and punitive measures associated with drug use (57), might preclude their reporting. Previous research also suggests that alcohol use is not associated with suicide in China (adjusted hazard ratio, 95% CI: 1.0, 0.8–1.3) (10). However, it is likely that substance misuse is associated with violence risk based on systematic reviews of the evidence, although this needs more work in middle income countries (58), including those with lower prevalences of substance misuse in patient populations. Considering that OxMIS and OxMIV were developed in Sweden and substance use is an important risk factor in Western countries (27, 28), further work can examine the effect of these risk factors in the Chinese population.

Both OxMIS and OxMIV include some mental health–related modifiable risk factors for managing risk of suicide and violence in people with severe mental illness, including recent antipsychotic, antidepressant or dependence treatment, substance misuse, and depression comorbidity. These risk factors can be justified as they provide key targets for clinical intervention, from which people at increased risk could benefit (46). Additional research on novel approaches to risk assessment is required to incorporate more detailed monitoring of fluctuations in symptoms and dynamic risk factors, although this will likely improve needs management rather than risk prediction (59).

Future investigations should consider differential clinical and legal definitions in the context of China or Spain, as these are relevant variables when conducting cross–cultural implementation research. For instance, while the concept of dangerousness toward others is universal, what constitutes a clinically or legally relevant threshold will likely differ between jurisdictions.

## Conclusion

Overall, the findings support the acceptability and feasibility of implementing OxMIS and OxMIV in two different countries across patient groups. These risk assessment tools were considered to be scalable and simple to use, and with their face validity, this suggests their potential adoption into general psychiatric services. Few practical barriers to usability were identified, and acceptability was high amongst clinicians. These tools could be incorporated in routine patient assessment, as an adjunct to clinical judgement, to improve risk prevention and management. However, the potential impact of the probability risk score on clinical decisions needs to be examined in future studies and ongoing external validations could be incorporated into plans for local implementation. These tools should be used in conjunction with clinical judgement to support decision-making and improve consistency in risk assessments, rather than on their own, as they do not encompass the whole spectrum of individual risk factors for any particular patient.

## Data Availability Statement

The datasets presented in this article are not readily available because they are based on health records which potentially contain identifiable patient information. Requests to access the datasets should be directed to SF, seena.fazel@psych.ox.ac.uk.

## Ethics Statement

The studies involving human participants were reviewed and approved by Institutional Review Boards of all three participating hospitals, namely the Second Xiangya Hospital of Central South University (Changsha, China), Hospital Universitario Germans Trías i Pujol (Barcelona, Spain) and Hospital Universitario Virgen del Rocío (Sevilla, Spain). Written informed consent for participation was not required for this study in accordance with the national legislation and the institutional requirements.

## Author Contributions

SF and RY conceived and designed the study. JO and MC-R oversaw the data collection, data extraction and ethical approval process in China and Spain, respectively. GB had full access to all data and performed the quantitative analysis. JM and GB conducted the qualitative analyses jointly. GB drafted the manuscript, and revised it with SF and RY. All authors read and approved the final manuscript.

## Funding

GB was funded by the Fonds de recherche du Québec – Santé (FRQS), grant number 282526. SF was funded by the Wellcome Trust, grant number 202836/Z/16/Z.

## Conflict of Interest

SF was part of the study team that first developed OxMIV and OxMIS. He has not received any compensation in relation to their development, use or translation. The remaining authors declare that the research was conducted in the absence of any commercial or financial relationships that could be construed as a potential conflict of interest.

## Publisher's Note

All claims expressed in this article are solely those of the authors and do not necessarily represent those of their affiliated organizations, or those of the publisher, the editors and the reviewers. Any product that may be evaluated in this article, or claim that may be made by its manufacturer, is not guaranteed or endorsed by the publisher.
