# Supplementary material for: Evaluating the Risk of Suicide and Violence in Severe Mental Illness: A Feasibility Study of Two Risk Assessment Tools (OxMIS and OxMIV) in General Psychiatric Settings
Source: Front Psychiatry. 2022 Jun 30;13:871213. doi: 10.3389/fpsyt.2022.871213 (PMC9280292; doi:10.3389/fpsyt.2022.871213)
Supplement: Supplementary file 1 [file Data_Sheet_1.docx]

**Supplementary Table 1.** Previously used tools or process for suicide and violence risk assessment as reported by clinicians in China and Spain

|  | **Risk assessment tools or process** | |
| --- | --- | --- |
|  | **China** | **Spain** |
| **Suicide** | Admission risk assessment (undefined)  Beck Scale for Suicide Ideation (BSS) (1)  Depression and mania scale (undefined)  Minnesota Multiphasic Personality Inventory–2 (MMPI–2) (2)  Psychiatric Risk Assessment Scale (PRAS) (3)  Risk assessment of suicide and impulsivity in hospital (undefined)  Sheehan Disability Scale (SDS) (4)  Suicide and Aggression Survey (5)  Suicide risk assessment scale (undefined)  Suicide risk behavior assessment scale (undefined) | Columbia – Suicide Severity Rating Scale (C–SSRS) (6)  Depression scale (undefined)  Plutchik Suicide Risk Scale (7)  SAD PERSONS Scale (SPS) (8) |
| **Violence** | Admission risk assessment (undefined)  Aggressive Behavior Risk Assessment Tool (ABRAT) (9)  Modified Overt Aggressive Behavior Scale (MOAS) (10)  Psychiatric Risk Assessment Scale (PRAS) (3)  Psychiatric violence risk assessment scale (undefined)  Risk assessment of suicide and impulsivity in hospital (undefined)  Risk Assessment Scale (undefined) | None |

**Supplementary Table 2.** OxMIS clinician interview results in China and Spain

| **Variable** | **China** (*N*= 26 clinicians) | **Spain** (*N*= 12 clinicians) |
| --- | --- | --- |
| *Risk of suicide (compared to the average annual rate)* | | |
| Higher than average | 10 | 7 |
| Around average | 22 | 4 |
| Below the average | 27 | 1 |
| Unable to answer | 1 |  |
| *Patient death by suicide* | | |
| Yes | 0 | 1 |
| No | 59 | 11 |
| Unknown | 1 | 0 |
| *OxMIS score accurate representation of risk* | | |
| Yes | 25 | 11 |
| No | 11 | 1 |
| Unable to say | 24 | 0 |
| *OxMIS score of clinical benefit* | | |
| Yes | 50 | 10 |
| No | 10 | 2 |
| *Profession* | | |
| Consultant psychiatrist | 22 | 11 |
| Clinical psychologist | 0 | 1 |
| Psychiatric nurse | 0 | 0 |
| Occupational therapist | 0 | 0 |
| Social worker | 0 | 0 |
| Doctor in training | 0 | 0 |
| Other | 4 | 0 |
| *Other routinely used tool or process for suicide risk assessment* | | |
| Yes | 36 | 5 |
| No | 24 | 7 |
| *Practical tool to use* | | |
| Yes | 49 | 12 |
| No | 11 | 0 |
| *Relevancy of risk factors* |  |  |
| Not at all relevant | 0 | 0 |
| Slightly relevant | 8 | 1 |
| Somewhat relevant | 34 | 1 |
| Very relevant | 16 | 10 |
| Extremely relevant | 2 | 0 |
| *Availability of the clinical information required* | | |
| Not at all available | 0 | 0 |
| Slightly available | 4 | 1 |
| Somewhat available | 27 | 2 |
| Very available | 27 | 4 |
| Extremely available | 2 | 4 |
| *Possible to complete without notes* | | |
| Yes | 24 | 8 |
| No | 36 | 4 |
| *Rating of OxMIS compared to clinical judgement of risk* | | |
| Not at all similar | 3 | 0 |
| Slightly similar | 4 | 0 |
| Somewhat similar | 33 | 3 |
| Very similar | 19 | 8 |
| Extremely similar | 1 | 1 |
| *OxMIS risk rating more often higher or lower compared to clinical judgement* | | |
| OxMIS rating higher | 29 | 10 |
| OxMIS rating lower | 31 | 2 |
| *Use of OxMIS in the future* | | |
| Yes | 54 | 10 |
| No | 6 | 2 |

Note. For the Chinese sample, 26 clinicians completed one questionnaire for each of the 60 patients. In Spain, each of the 12 clinicians completed the questionnaire for one patient only.

**Supplementary Table 3.** OxMIV clinician interview results in China and Spain

| **Variable** | **China** (*N*= 26 clinicians) | **Spain** (*N*= 12 clinicians) |
| --- | --- | --- |
| *Profession* | | |
| General psychiatrist | 22 | 11 |
| Forensic psychiatrist | 0 | 0 |
| Clinical psychologist | 0 | 1 |
| Psychiatric nurse | 0 | 0 |
| General practitioner | 2 | 0 |
| Other | 2 | 0 |
| *Clinical setting (multiple answers allowed)* | | |
| Psychiatric inpatient in a general adult ward | 32 | 7 |
| Psychiatric intensive care unit | 37 | 0 |
| Secure/forensic psychiatric hospital | 4 | 0 |
| Community psychiatry | 4 | 5 |
| Prison | 4 | 0 |
| Part of a multidisciplinary team meeting/referrals meeting or equivalent | 4 | 0 |
| Other | 8 | 0 |
| *Other routinely used tool or process for violence risk assessment* | | |
| Yes | 23 | 0 |
| No | 37 | 12 |
| *Any preconceptions about the usefulness of a simple risk assessment tool for violence* | | |
| None | 28 | 5 |
| Not useful | 0 | 1 |
| Slightly useful | 11 | 2 |
| Somewhat useful | 19 | 2 |
| Very useful | 2 | 2 |
| Extremely useful | 0 | 0 |
| *Number of patients* |  |  |
| 1–4 | 25 | 0 |
| 5–9 | 0 | 5 |
| 10–20 | 0 | 7 |
| >20 | 1 | 0 |
| Extremely relevant | 2 | 0 |
| *Availability of the clinical information required* | | |
| Not at all available | 0 | 0 |
| Slightly available | 4 | 0 |
| Somewhat available | 36 | 4 |
| Very available | 20 | 6 |
| Extremely available | 0 | 2 |
| *Frequency of complete information (i.e. without entering ‘unknown’ for any item)* | | |
| Not at all frequently | 6 | 0 |
| Slightly frequently | 15 | 0 |
| Somewhat frequently | 15 | 3 |
| Very frequently | 19 | 7 |
| Extremely frequently | 5 | 2 |
| *Relevancy of the risk factors included* | | |
| Not at all relevant | 0 | 0 |
| Slightly relevant | 6 | 0 |
| Somewhat relevant | 30 | 2 |
| Very relevant | 24 | 8 |
| Extremely relevant | 0 | 2 |
| *General satisfaction with ease of use of the online calculator* | | |
| Not at all satisfied | 0 | 0 |
| Slightly satisfied | 9 | 0 |
| Somewhat satisfied | 17 | 3 |
| Very satisfied | 32 | 7 |
| Extremely satisfied | 2 | 2 |
| *Level of patient involvement in completing OxMIV* | | |
| Patient(s) not aware of use of OxMIV | 34 | 6 |
| Patient(s) aware of use of OxMIV but not involved in completion or discussion of score | 19 | 2 |
| Patient(s) not involved in completion but informed of score | 4 | 1 |
| Patient(s) directly involved in OxMIV completion and discussion of score | 3 | 3 |
| *Helpfulness of patient involvement* | | |
| Not applicable | 1 | 6 |
| Not at all helpful | 2 | 0 |
| Slightly helpful | 10 | 1 |
| Somewhat helpful | 30 | 1 |
| Very helpful | 15 | 4 |
| Extremely helpful | 2 | 0 |
| *Patient acceptability in completing OxMIV or discussing the score* | | |
| Not applicable | 1 | 6 |
| Not at all acceptable | 2 | 0 |
| Slightly acceptable | 10 | 1 |
| Somewhat acceptable | 30 | 1 |
| Very acceptable | 15 | 4 |
| Extremely acceptable | 2 | 0 |
| *Rating of OxMIV compared to clinical judgement of risk* | | |
| Not at all similar | 1 | 0 |
| Slightly similar | 6 | 0 |
| Somewhat similar | 40 | 2 |
| Very similar | 12 | 9 |
| Extremely similar | 1 | 1 |
| *OxMIV risk rating more often higher or lower compared to clinical judgement* | | |
| OxMIV rating higher | 28 | 8 |
| OxMIV rating lower | 32 | 4 |
| *Impact of OxMIV when it differed from clinical judgement* | | |
| No, I tended to stick with my original judgement | 13 | 3 |
| Yes, I tended to alter my overall judgement to be closer to OxMIV | 13 | 6 |
| I tended to go with whichever rated risk higher | 34 | 3 |
| I tended to go with whichever rated risk lower | 0 | 0 |
| *Helpfulness of thinking more about risk when OxMIV and clinical judgement differed* | | |
| Not applicable | 0 | 0 |
| Not at all helpful | 0 | 0 |
| Slightly helpful | 1 | 2 |
| Somewhat helpful | 13 | 6 |
| Very helpful | 31 | 3 |
| Extremely helpful | 15 | 1 |
| *Most helpful OxMIV output* | | |
| Percentage score | 13 | 1 |
| Categories (low vs. increased) | 7 | 7 |
| Both combined | 40 | 4 |
| *OxMIV had a differential impact on clinical practice* | | |
| Yes | 24 | 6 |
| No | 36 | 6 |
| *Practical barriers in local clinical setting that limited the use of OxMIV* | | |
| Yes | 4 | 1 |
| No | 56 | 11 |
| *Problems arising from using OxMIV* | | |
| Yes | 8 | 0 |
| No | 52 | 12 |
| *Usefulness to clinical practice* | | |
| Not at all useful | 0 | 1 |
| Slightly useful | 9 | 0 |
| Somewhat useful | 40 | 6 |
| Very useful | 11 | 5 |
| Extremely useful | 0 | 1 |
| *Likelihood of continuing use of OxMIV in the future* | | |
| Not at all likely | 0 | 1 |
| Slightly likely | 8 | 0 |
| Somewhat likely | 40 | 6 |
| Very likely | 11 | 5 |
| Extremely likely | 1 | 1 |

Note. For the Chinese sample, 26 clinicians completed one questionnaire for each of the 60 patients. In Spain, each of the 12 clinicians completed the questionnaire for one patient only.

**REFERENCES**

1. Beck AT, Kovacs M, Weissman A. Assessment of Suicidal Intention: The Scale for Suicide Ideation. *J Consult Clin Psychol* (1979) 47(2):343-52. doi: 10.1037//0022-006x.47.2.343.

2. Butcher JN, Atlis MM, Hahn J. The Minnesota Multiphasic Personality Inventory-2 (MMPI-2). *Comprehensive Handbook of Psychological Assessment, Vol 2: Personality Assessment*. Hoboken, NJ, US: John Wiley & Sons, Inc. (2004). p. 30-8.

3. Shoka A, Lazzari C. Psychiatric Risk Assessment Scale (PRAS). *Eur Psychiatry* (2017) 41(S1):S731-S2. doi: 10.1016/j.eurpsy.2017.01.1338.

4. Sheehan DV, Harnett-Sheehan K, Raj BA. The Measurement of Disability. *Int Clin Psychopharmacol* (1996) 11 Suppl 3:89-95. doi: 10.1097/00004850-199606003-00015.

5. Korn ML, Botsis AJ, Kotler M, Plutchik R, Conte HR, Finkelstein G, et al. The Suicide and Aggression Survey: A Semistructured Instrument for the Measurement of Suicidality and Aggression. *Compr Psychiatry* (1992) 33(6):359-65. doi: 10.1016/0010-440x(92)90056-v.

6. Posner K, Brown GK, Stanley B, Brent DA, Yershova KV, Oquendo MA, et al. The Columbia-Suicide Severity Rating Scale: Initial Validity and Internal Consistency Findings from Three Multisite Studies with Adolescents and Adults. *Am J Psychiatry* (2011) 168(12):1266-77. doi: 10.1176/appi.ajp.2011.10111704.

7. Plutchik R, van Praag HM, Conte HR, Picard S. Correlates of Suicide and Violence Risk 1: The Suicide Risk Measure. *Compr Psychiatry* (1989) 30(4):296-302. doi: 10.1016/0010-440x(89)90053-9.

8. Patterson WM, Dohn HH, Bird J, Patterson GA. Evaluation of Suicidal Patients: The SAD PERSONS Scale. *Psychosomatics* (1983) 24(4):343-5, 8-9. doi: 10.1016/s0033-3182(83)73213-5.

9. Kim SC, Ideker K, Todicheeney-Mannes D. Usefulness of Aggressive Behaviour Risk Assessment Tool for Prospectively Identifying Violent Patients in Medical and Surgical Units. *J Adv Nurs* (2012) 68(2):349-57. doi: 10.1111/j.1365-2648.2011.05744.x.

10. Sorgi P, Ratey J, Knoedler DW, Markert RJ, Reichman M. Rating Aggression in the Clinical Setting. A Retrospective Adaptation of the Overt Aggression Scale: Preliminary Results. *J Neuropsychiatry Clin Neurosci* (1991) 3(2):S52-6.
